# Supplementary material for: Three-dimensional architecture and linearized mapping of vibrissa follicle afferents
Source: Nat Commun. 2025 Jan 8;16:499. doi: 10.1038/s41467-024-55468-4 (PMC11711312; doi:10.1038/s41467-024-55468-4)
Supplement: Supplementary file 1 — Supplementary Information [file 41467_2024_55468_MOESM1_ESM.pdf]

Three-dimensional architecture and linearized mapping of vibrissa follicle afferents

Supplementary Information

Supplementary Table 1 | Follicle innervation similarities across animals

The second stained dataset contains only a partially (50%) visible C2 vibrissa follicle. The grey half-circle occluding the rostral half of the polarization plot refers to the missing part of this follicle dataset.

	Stained dataset	Stained dataset (50% reconstructed)	Unstained dataset	Average (mean ± SD)
**Total axons**	≥239	n.a.	n.a.	n.a.
**Myelinated axons**	171	177 (89 reconstructed)	174	174 ± 4
**Unmyelinated axons**	≥58	n.a.	n.a.	n.a.
**Afferents types**<br>Merkel<br>Lanceolate<br>Club like<br>Ruffini	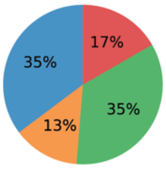	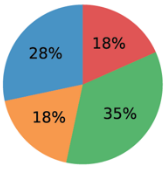	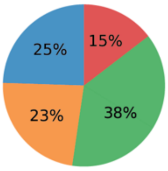	Merkel: 29 ± 5%<br>Lanceolate: 18 ± 5%<br>Club like: 36 ± 2%<br>Ruffini: 17 ± 2%
**Subtypes**	Merkel and Club like	n.a.	n.a.	n.a.
**Polarization**<br>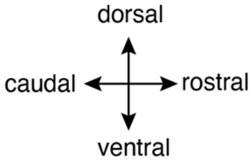	dorso-caudal<br>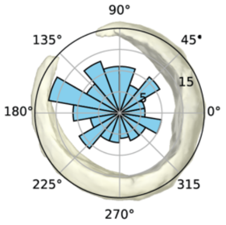	presumably dorso-caudal<br>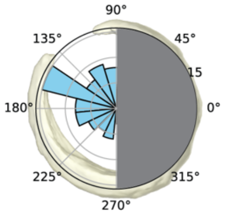	dorso-caudal<br>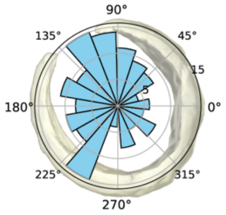	dorso-caudal<br>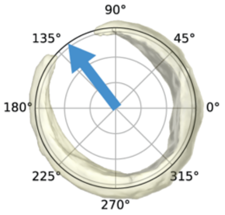
**Velocity gradient**	Proximal to distal	Proximal to distal	n.a.	Proximal to distal
**Linearization**	Yes	Yes	Yes	Yes

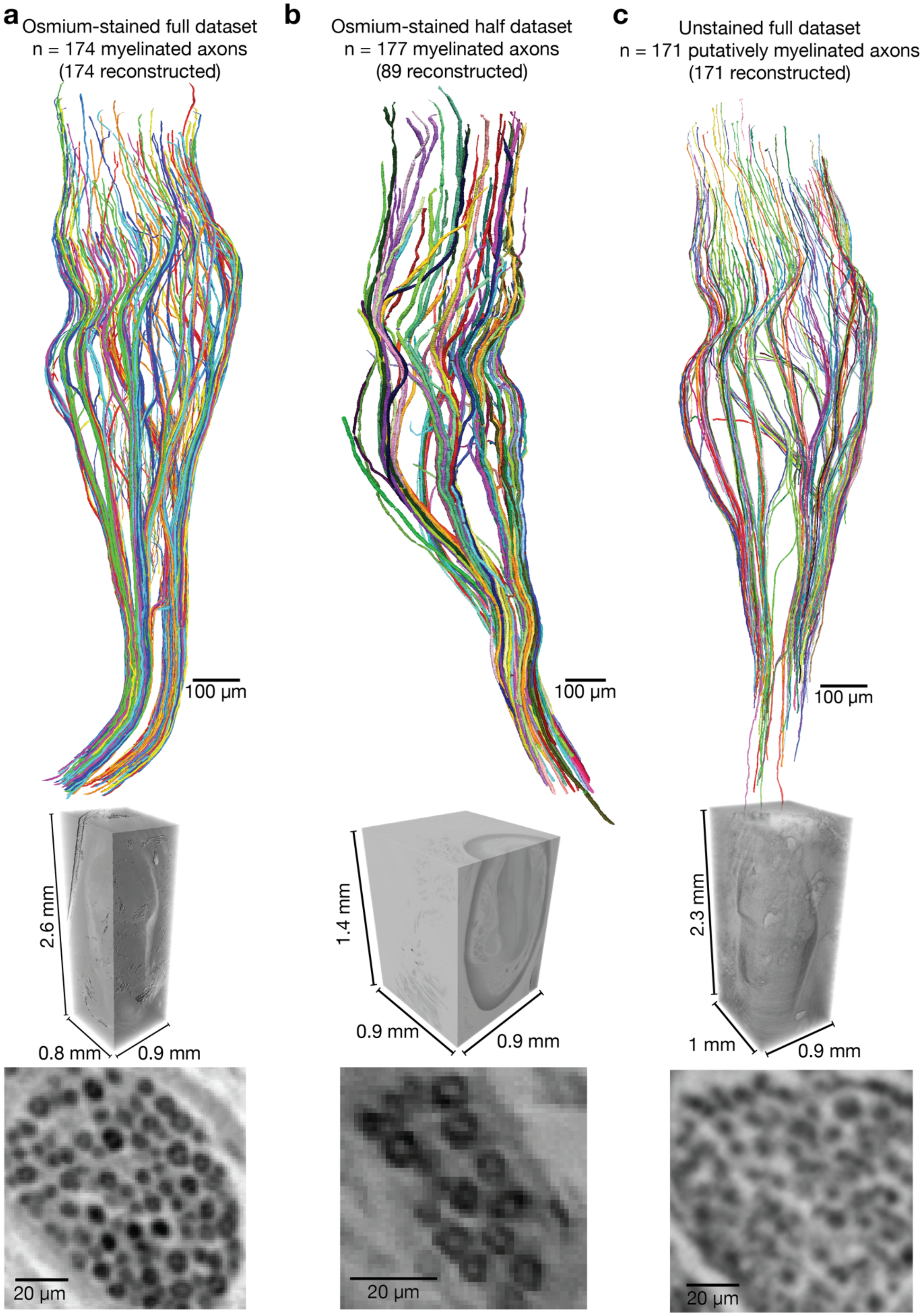
a
Osmium-stained full dataset
n = 174 myelinated axons
(174 reconstructed)
100 µm
2.6 mm
0.8 mm
0.9 mm
20 µm
b
Osmium-stained half dataset
n = 177 myelinated axons
(89 reconstructed)
100 µm
1.4 mm
0.9 mm
0.9 mm
20 µm
c
Unstained full dataset
n = 171 putatively myelinated axons
(171 reconstructed)
100 µm
2.3 mm
1 mm
0.9 mm
20 µm

Supplementary Fig. 1 | Myelin- and lumen annotated vibrissa follicle datasets.

a, Reconstruction of afferent innervation, raw image volume and nerve crossection from a complete, osmium-stained dataset. Axonal reconstruction is based on myelinated fiber segmentation.

b, As **a** but for a partially incomplete, osmium-stained dataset.

c, As **a**, but for an unstained dataset. Axonal reconstruction is based on lumen segmentation of putatively myelinated fibers.

Supplementary Fig. 2 | Predominance of vertical and shaft-aligned axonal trajectories.

a, Boxplot of average X, Y and Z trajectories per afferent. Z trajectories display highest magnitude with only little variance. Boxplots display the 25th to 75th percentile range as the box and the median as center line. Boxplot whiskers extend by the inter quartile range. Outliers are plotted individually.

b, Histogram of average z-trajectory vector angles relative to the vibrissa shaft.

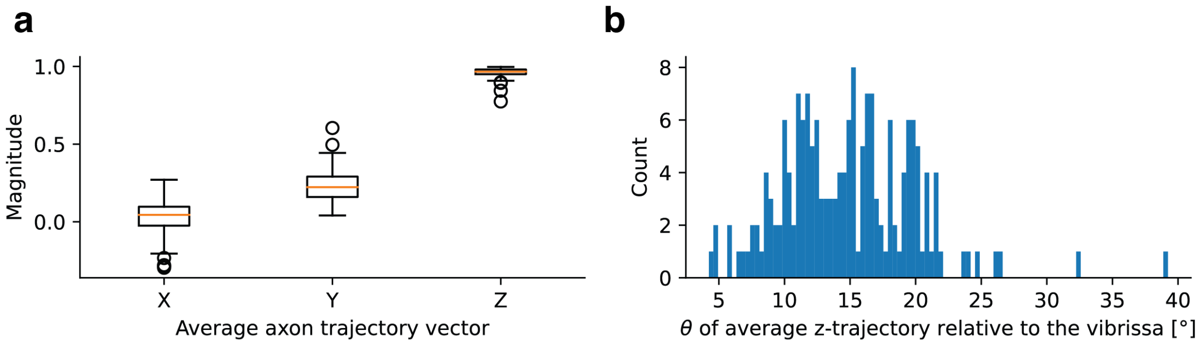

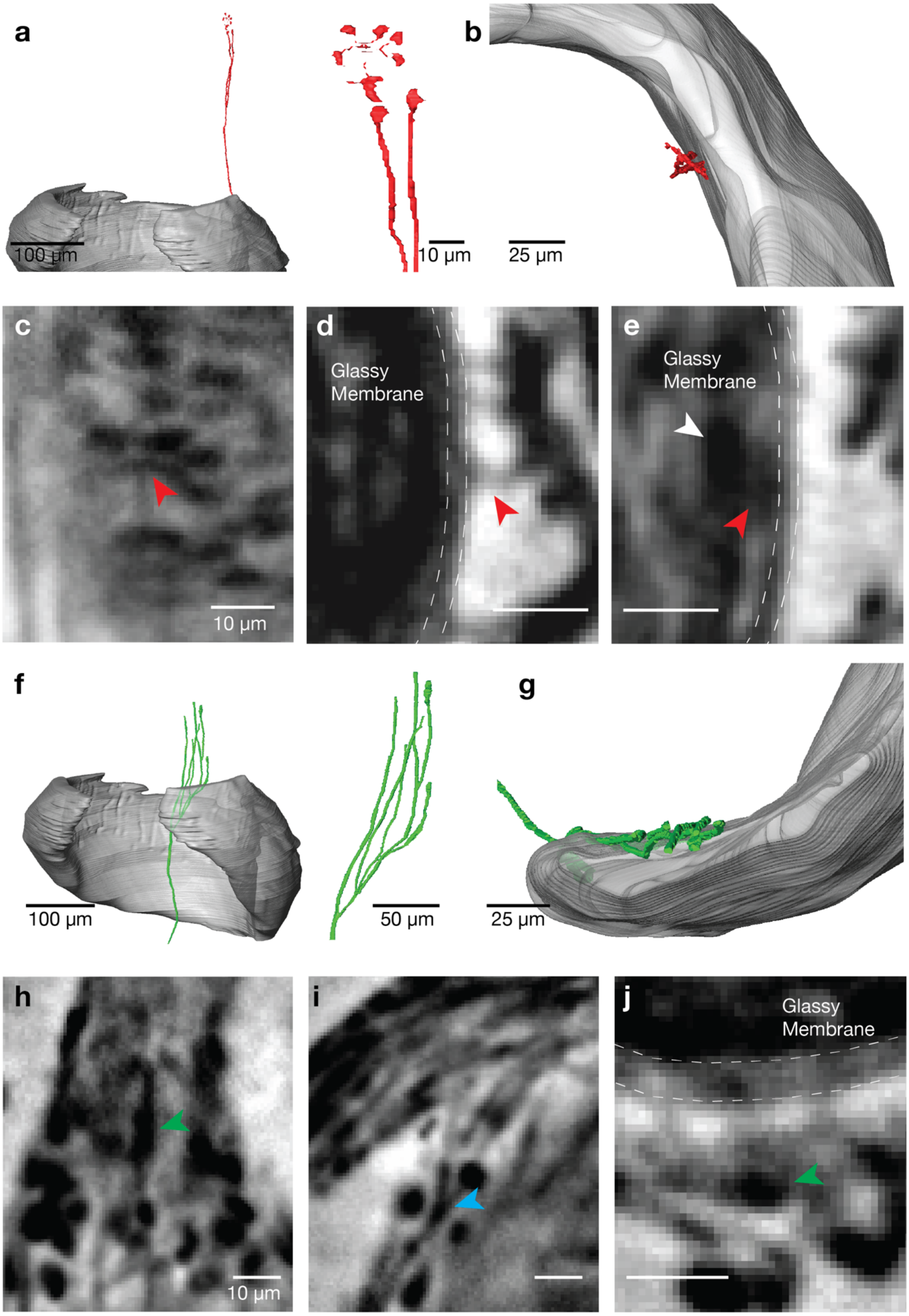

a
100 µm
10 µm
b
25 µm
c
10 µm
d
Glassy Membrane
e
Glassy Membrane
f
100 µm
50 µm
g
25 µm
h
10 µm
i
j
Glassy Membrane

Supplementary Fig. 3 | Fine structure of Merkel and lanceolate afferents.

a, Left: Side view of a volume rendering of a completely reconstructed Merkel afferent and ringwulst. Right: High magnification of the Merkel ending.

b, Top view of a volume rendering of the completely reconstructed Merkel afferent and ringwulst.

c, 2D virtual section side view showing the foot-like endings of the Merkel afferent. Red arrow indicates the axon connecting to the Merkel afferent.

d, 2D virtual section top view showing the root sheath entry of the Merkel afferent (red arrow); this is the distinguishing feature of all Merkel afferents. The dotted line indicates the glassy membrane (applies to all panels).

e, 2D virtual section (slightly superior to d) top view showing a Merkel afferent foot (red arrow) adjacent to a putative Merkel cell (X-ray dense structure indicated by the white arrow).

f, Left: Side view of a volume rendering of a completely reconstructed lanceolate-afferent and ringwulst. Right: High magnification of the lanceolate ending.

g, Top view of a volume rendering of the completely reconstructed lanceolate afferent and ringwulst.

h, 2D virtual section side view showing the spear-like lanceolate ending (green arrow).

i, 2D virtual section side view showing a branching point of the lanceolate afferent (blue arrow).

j, 2D virtual section top view showing the ending point of one lanceolate afferent branch (green arrow); the ending of these afferents outside of the root sheath adjacent to the glassy membrane was the distinguishing feature for all lanceolate afferents.

Lanceolate endings could usually be completely reconstructed. The thin feet of Merkel-cell endings, however, could not always be connected to the Merkel afferent entering the root sheath. We therefore only show the root sheath entries of the Merkel afferent in Fig. 3b.

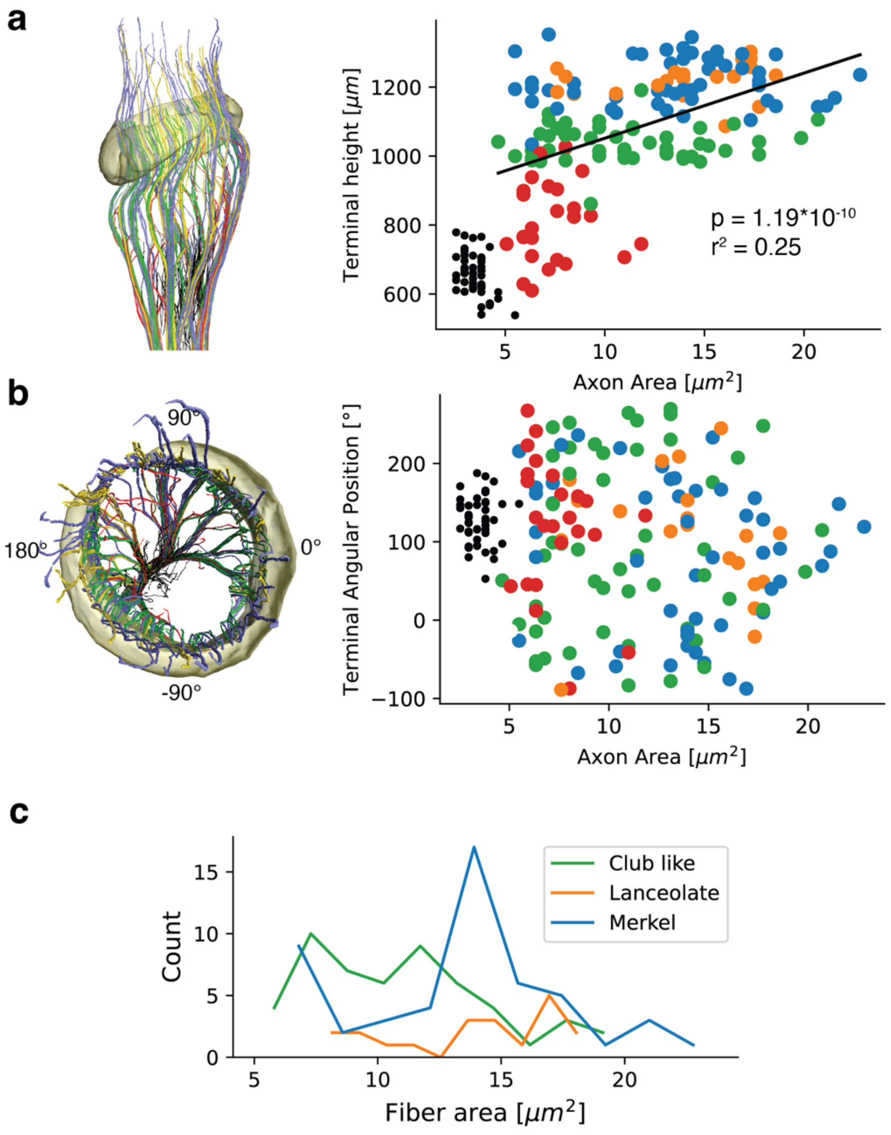

Supplemetary Fig. 4 | Axon diameter gradient by terminal height and angular position across ending types.

a, Left: Deep vibrissal afferents color coded by afferent type (Merkel = blue, lanceolate = orange, club = green, Ruffini = red, unmyelinated = black). Right: Axon area as a function of afferent terminal height (R^2 = 0.25, p = 2.72 * 10^{-10} for myelinated data only).

b, Left: Top view of deep vibrissal afferents color coded by afferent type (color conventions as in **a**). Right: Axon areas scatter equally across the positions of radial afferents, with a slight peak between 90 – 180° positioned afferents.

c, Histogram of fiber areas of Club like (green), lanceolate (orange) and Merkel (blue) afferents.
